# Comparative Evaluation of Immune Responses and Protection of Chitosan Nanoparticles and Oil-Emulsion Adjuvants in Avian Coronavirus Inactivated Vaccines in Chickens

**DOI:** 10.3390/vaccines9121457

**Published:** 2021-12-09

**Authors:** Priscila Diniz Lopes, Cintia Hiromi Okino, Filipe Santos Fernando, Caren Pavani, Viviane Casagrande Mariguela, Maria de Fátima Silva Montassier, Hélio José Montassier

**Affiliations:** 1 Department of Veterinary Pathology, School of Agricultural and Veterinarian Sciences, Jaboticabal, São Paulo State University (Unesp), Jaboticabal 14884-900, Brazil; filipe.fernando@yahoo.com.br (F.S.F.); caren_pavani@hotmail.com (C.P.); vivi.mariguela@gmail.com.br (V.C.M.); fatimamontassier@gmail.com (M.d.F.S.M.); helio.montassier@unesp.br (H.J.M.); 2 Embrapa Southeast Livestock, Brazilian Agricultural Research Corporation (Embrapa), Canchim Farm, São Carlos 13560-970, Brazil; cintia.okino@embrapa.br

**Keywords:** avian infectious bronchitis, cellular immune response, delivery carrier-adjuvant, mucosal immunization, oil adjuvant

## Abstract

Efficient vaccines are the main strategy to control the avian coronavirus (AvCoV), although several drawbacks related to traditional attenuated and inactivated vaccines have been reported. These counterpoints highlight the importance of developing new alternative vaccines against AvCoV, especially those able to induce long-lasting immune responses. This study evaluated and compared two inactivated vaccines formulated with AvCoV BR-I variants, one composed of chitosan nanoparticles (AvCoV-CS) and the second by Montanide oily adjuvant (AvCoV-O). Both developed vaccines were administered in a single dose or associated with the traditional Mass attenuated vaccine. The AvCoV-CS vaccine administered alone or associated with the Mass vaccine was able to induce strong humoral and cell-mediated immune (CMI) responses and complete protection against IBV virulent infection, wherein single administration was characterized by high IgA antibody levels in the mucosa, whereas when associated with the Mass vaccine, the serum IgG antibody was predominantly observed. On the other hand, single administration of the oily vaccine presented poor humoral and CMI responses and consequently incomplete protection against virulent challenge, but when associated with the Mass vaccine, immune responses were developed, and complete protection against infection was observed. Both of our experimental vaccines were able to induce full protection against virulent IBV challenge. A single dose of AvCoV-CS vaccine was sufficient to achieve complete protection, while AvCoV-O required a previous priming by a Mass strain to complete the protection.

## 1. Introduction

Live attenuated and inactivated vaccines, containing the most epidemiologically important strains of the avian coronavirus (AvCoV), have been extensively used for the control of infectious bronchitis (IB) in poultry. However, despite the constant increase in new vaccines, there are still several reports of incomplete protection against emerging new variants, mainly due to poor cross-protective immune responses developed in the respiratory mucosa, which is the portal of entry for this virus [1,2,3,4]. In addition, the continuous emergence of AvCoV variants, even in vaccinated flocks, is derived from high mutation and recombination rates that are driven by selective pressures exerted by a partial immune status of avian hosts [5,6,7,8,9].

AvCoV inactivated vaccines with oil adjuvant are commonly used in layer and breeder chickens by intramuscular or subcutaneous injections but are not usually administered to broilers [10]. The vaccine response is characterized by high systemic anti-AvCoV antibody titers while poor T cytotoxic lymphocyte activation and local humoral responses are induced, consequently resulting in incomplete protection against AvCoV infection [5,9]. In turn, inactivated vaccines are considered as safe, since inactivated viruses are not capable of replicating, causing reversion of virulence, or gene recombination, as well as reactivating residual pathogenicity. Therefore, alternative inactivated antigenic delivery and adjuvant systems are required, especially for those targeting the enhancement of the immunogenic responses. In this context, new adjuvants and/or carrier systems for AvCoV inactivated vaccines may constitute relevant approaches to replace traditional adjuvants, well known to be associated with increased toxicity, lower immunogenicity, and adverse reactions [11,12].

Chitosan nanoparticles (CPS) have been recognized as promising carrier-adjuvant candidates for mucosal stimulation [12,13,14,15,16] and are able to drive both cell-mediated (CMI) and humoral immune responses in mucosal and systemic compartments [12,13,15,16]. Thus, CPSs have been successfully used as delivery carrier-adjuvants for several antigens and DNA preparations in diverse vaccine formulations [12,13,15,17,18,19]. In addition, the association of CPS and inactivated AvCoV has already proven to be able to induce humoral and CMI responses at the primary site of AvCoV replication, which were correlated with decreased pathological lesions and viral loads [20], similarly to the protection afforded by live attenuated AvCoV vaccines [1,2,3].

Montanide ISA 71 from the oil adjuvants class is a refined mineral oil less viscous and more stable than Freund-type adjuvants. This oil adjuvant was developed to improve both T_H1_ response and immunoglobulin (Ig) G2 production, even for antigens presenting low intrinsic immunogenicity levels [21]. Montanide ISA 71 has been used to formulate experimental vaccines composed of recombinant proteins against *Eimeria* spp. and avian influenza virus. Several studies have demonstrated that these formulations induced prominent antibody and cell-mediated immune responses in chickens [22,23]. In addition, Montanide ISA 71 was also successfully used to formulate an inactivated BR-I AvCoV vaccine, administered as a booster after a priming dose of the attenuated Massachusetts (Mass) vaccine in specific pathogen-free (SPF) chickens. This combination was able to elicit strong systemic and local immune responses, resulting in effective protection against the BR-I AvCoV variant [24].

Finally, the use of CPS and Montanide ISA 71 as carrier-adjuvants in poultry vaccines has been proved to induce the development of complete protection against clinical disease. However, there are no comparative studies reporting the effect of these adjuvants associated with inactivated AvCoV against IB; furthermore, different routes of administration remain to be evaluated [15,20,22,23,25,26]. The aim of this study was to compare the vaccine efficacy of an inactivated AvCoV Brazilian variant strain (lineage 11 of genotype I, formerly classified as BR-I genotype) encapsulated in chitosan nanoparticles administered by the mucosal route versus that incorporated into the oil adjuvant Montanide ISA 71 administered by intramuscular injection.

## 2. Materials and Methods

### 2.1. Virus

The IBV/Brazil/PR05 AvCoV strain (GenBank: MK957244.1) was replicated in a 10-day-old specific pathogen free (SPF) embryonated chicken egg system, followed by determination of the infectivity titer of the harvested allantoic fluid (AF) suspension in the same system, as recommended [27]. The viral AF suspension containing a 10^8.285^ embryo infectious dose (EID50)/mL was inactivated by beta-propiolactone at a concentration of 1:2000 for 90 min at 37 °C with continuous stirring, followed by checking the virus inactivation in SPF-embryonated chicken eggs [28]. The AF containing the inactivated AvCoV was stored at −70 °C until be processed.

### 2.2. Preparations of Vaccines

#### 2.2.1. AvCoV-CS

The encapsulation of inactivated BR-I AvCoV virions in chitosan nanoparticles (AvCoV-CS) was produced by an ionic gelation method as reported [20]. In brief, 0.6 mL of the virus in allantoic fluid (AF) was added dropwise to 5 mL of a solution of 0.05% chitosan (medium-weight molecular; Sigma-Aldrich, St. Louis, MO, USA) at maximum stirring. Then, 1 mL of 0.1% sodium tripolyphosphate (Sigma-Aldrich, St. Louis, MO, USA) solution was added dropwise to the solution under magnetic stirring and incubated for 10 min at room temperature.

#### 2.2.2. AvCoV-O

The AvCoV-O vaccine was prepared (*v*/*v*) by incorporating 70% of the adjuvant Montanide ISA 71 (SEPPIC, Inc., Fairfield, CT, USA) and 30% of the virus in AF to obtain a water-in-oil emulsion [24].

#### 2.2.3. H120 Attenuated Vaccine (L)

A lyophilized commercial attenuated vaccine containing the H120 strain of AvCoV was prepared in Dulbecco’s modified Eagle’s medium (DMEM, Gibco, Thermo Fisher Scientific, Waltham, MA, USA) to obtain the indicated dose.

### 2.3. In Vivo Vaccine Evaluation

A set of 162 SPF day-old White Leghorn chickens was randomly separated into six experimental groups (30 chickens per each vaccinated group and 21 chickens for each control group), which were placed in six positive-pressure isolators (Table 1). All groups were housed in separated positive-pressure isolators. On the first day of age, the chickens from the L+Nano (*n =* 30) and L+Oil groups (*n =* 30) were vaccinated with the H120 vaccine via the oculonasal route. At 14 days of age, chickens from the L+Nano and Nano groups (*n =* 30) received 100 μL of AvCoV-CS (10^8.285^ EID50 of AvCoV) via the oculonasal route, while chickens from the L+Oil and Oil groups (*n =* 30) received 300 μL of AvCoV-O (10^8.285^ EID50 of AvCoV) via the intramuscular route (pectoral region). At 31 days of age, the vaccinated groups and a nonvaccinated group (NV group, *n =* 21) were challenged with 10^4.5^ EID50/bird of a BR-I virulent strain of AvCoV via the oculonasal route. Another group (NC group, *n =* 21) was mock vaccinated with 100 μL of chitosan-free nanoparticles, with no virus at 14 days, and received 100 μL of Dulbecco’s modified Eagle’s medium on the challenge day (31 days of age), both via the oculonasal route. Serum and tear samples were collected from the chickens at the pre-challenge period (1 day preinfection) and at 1, 5, and 11 days post infection (dpi). Chickens from all experimental groups were randomly euthanized at 1, 5, and 11 dpi and submitted to tissue sample collections.

#### 2.3.1. Microscopic Alterations

Trachea and kidney samples collected at 5 and 11 dpi were processed by conventional histologic techniques, including hematoxylin and eosin staining for histopathology examination. The microscopic lesions for the trachea and kidney were evaluated by scores ranging from 0 to 3 (0 = no lesions, 1 = mild lesions, 2 = moderate lesions, 3 = severe lesions), as previously recommended [3,29,30,31,32].

#### 2.3.2. RNA Extraction

All the procedures for tissue collection and storage of trachea (collected at 1, 5, and 11 dpi) and kidney (collected at 5 and 11 dpi) samples from experimentally infected chickens and the processing for RNA extraction, quantification, and quality analysis followed the descriptions by Lopes et al. [20]. In summary, the RNA was extracted by a combination of QIAzol Lysis Reagent (Qiagen, Crawley, West Sussex, UK) and the RNeasy Mini Kit (Qiagen, Crawley, West Sussex, UK). The extracted RNA was quantified by spectrophotometry at 260 nm, and the quality was evaluated by spectrophotometry at 260 and 280 nm ratio and by agarose gel electrophoresis or by the Agilent RNA 6000 Nano Kit (Agilent Technologies, South Queensferry, UK). The RNA was stored at −70 °C until processing.

#### 2.3.3. Quantification of AvCoV Load

The viral loads were determined in RNA preparations extracted from tissue samples of experimentally infected chickens by the RT-qPCR technique. The primers and probe specific for a 3′-UTR of the AvCoV genome were used in conjunction with the reagents of the AgPath-IDTM One-Step RT-PCR Kit (Ambion, Applied Biosystems, Foster City, CA, USA) as previously described [33]. The mix preparation and reaction profiles were previously standardized by Lopes et al. [20]. All samples were run in duplicates. Linear regression analysis between the number of RNA copies and cycle quantification (Cq) was determined from a ten-fold dilution series of a standard and purified AvCoV RNA preparation to relate the Cq found for each tested tissue sample with the number of RNA copies [33].

#### 2.3.4. Quantification of the Expression of Cell-Mediated Immune (CMI) Response Genes

The expression of CMI genes in tissue samples was evaluated by the RT-qPCR technique as recommended by Okino et al. [32]. The extracted RNA from tissue samples was reverse-transcribed to synthesize cDNA using Oligo-DT primers and a Moloney Murine Leukemia Virus RT kit (Thermo Fisher Scientific, Waltham, MA, USA) according to the manufacturer’s instructions. The cDNA was submitted to qPCR with SYBR Green I to evaluate the relative expression of CMI genes, using previously described primers for CD8β and Granzyme A genes [20,32] and a new pair of primers for the Perforin-1 gene (Forward: ACTGCGTGCACTACTGGATCTC and Reverse: GGATGTAGTGGGTGCCGTATG). The reaction was performed using the QuantiFast SYBR Green PCR Kit (Qiagen, Crawley, West Sussex, UK) and real-time PCR equipment (7500, Applied Biosystems, Foster City, CA, USA). The Cq values obtained in qPCR for the CD8β, Granzyme A, and Perforin-1 genes were normalized using two reference genes (*GAPDH* and *TOP2B*) [20,32], and their levels of expression were calculated as fold changes related to the samples from nonvaccinated and nonchallenged chickens (NC group) [34].

#### 2.3.5. Evaluation of Mucosal and Systemic Anti-AvCoV Antibody Responses

Lachrymal secretion and serum samples were tested for quantification of the levels of anti-AcCoV antibodies of IgA and IgG isotypes, respectively, using the S-ELISA-Concanavalin A technique performed according to Bronzoni et al. [35]. Briefly, single dilutions of 1:25 or 1:50 in PBS of lachrymal or serum samples, respectively, were tested in duplicate, and the remaining reagents, such as Concanavalin A, AvCoV IBV/PR05 antigen suspension, anti-chicken IgA, and anti-chicken IgG peroxidase conjugates (Bethyl Laboratories, Montgomery, AL, USA), were used as recommended Bronzoni et al. [35] and Lopes et al. [20]. The anti-AvCoV IgA and IgG antibody levels were evaluated as sample-to-positive (S/P) values, which were calculated, following the recommendations from Bronzoni et al. [35] and Santos et al. [24], from the differences between the optical densities (ODs) of each tested lachrymal or serum samples and the ODs of negative and positive reference samples for lachrymal secretion or serum and for the presence of anti-AvCoV IgA or IgG antibodies, respectively.

### 2.4. Statistical Analysis

Statistical analyses were performed using Prism v.6.0 software (GraphPad Software, Inc., La Jolla, CA, USA), and the tests of Kruskal–Wallis and Dunn tests were used to compare the data from viral loads, scores of microscopic lesions, levels of IgA and IgG anti-AvCoV antibodies, and values of expression of cell-mediated immune response genes between the experimental groups of vaccinated and nonvaccinated chickens submitted to challenge. The correlations between the data of humoral and cell-mediated immune responses and the pathology changes (AvCoV loads) were estimated using the Spearman test. The probability level for significance was considered *p* ≤ 0.05 for all statistical analyses.

## 3. Results

### 3.1. Microscopic Alterations

The most frequent microscopic changes observed in tracheal samples were loss of cilia (deciliation) of epithelial cells, presence of lymphocytic cell infiltrates, and degeneration of mucous glands and congestion, while in some samples, loss of epithelial cells and epithelial hyperplasia were also observed (Supplementary Figure S1). In renal tissue, the most frequent lesions were degeneration and necrosis of tubular cubic cells and mononuclear inflammation (Supplementary Figure S1). Lower scores of microscopic lesions for the trachea and kidney were observed in chickens from the Nano, L+Nano, and L+Oil-vaccinated groups compared to the nonvaccinated (NV) and Oil-vaccinated groups (*p* ≤ 0.05) at 5 and 11 dpi (Table 2). There was no relevant microscopic alteration in all evaluated tissues from the NC group for all intervals.

### 3.2. Quantification of AvCoV Load in Tissue Samples

**Figure 1:**
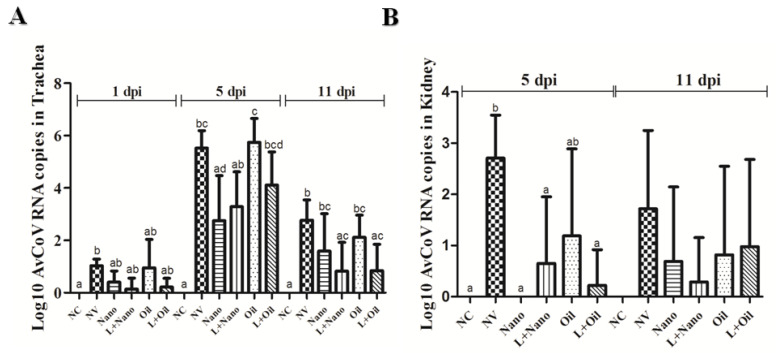
Means (± standard deviation) of Log10 AvCoV RNA copies detected in tracheal (**A**) and renal (**B**) samples observed at 1, 5, and 11 dpi of negative control (NC) group, nonvaccinated and challenged (NV) group, vaccinated with AvCoV-CS and challenged (Nano) group, vaccinated with H120 strain and AvCoV-CS and challenged (L+Nano) group, vaccinated with AvCoV-O and challenged (Oil) group, and vaccinated with H120 strain and AvCoV-O and challenged (L+Oil) group. Significantly (*p* ≤ 0.05) different means are shown as different letters.

The results of the means and standard deviations of the viral loads are presented in Figure 1. The viral loads found in tracheal samples were low at 1 dpi for all the challenged groups, including vaccinated and nonvaccinated birds. At 5 dpi, the Nano- and L+Nano-vaccinated groups had lower viral loads compared to the Oil group. NV also differed from the Oil group (*p* ≥ 0.05). The viral loads declined at 11 dpi, but the nonvaccinated (NV) chickens still showed higher viral loads when compared to those detected in the L+Nano and L+Oil groups (*p* ≤ 0.05).

For the renal samples, the Nano-, L+Nano-, and L+Oil-vaccinated groups showed significantly lower viral loads at 5 dpi (*p* ≤ 0.05), while the Oil group had no significant difference compared to the NV group. No significant difference was observed for the renal viral loads between the groups at 11 dpi.

### 3.3. CMI Responses

**Figure 2:**
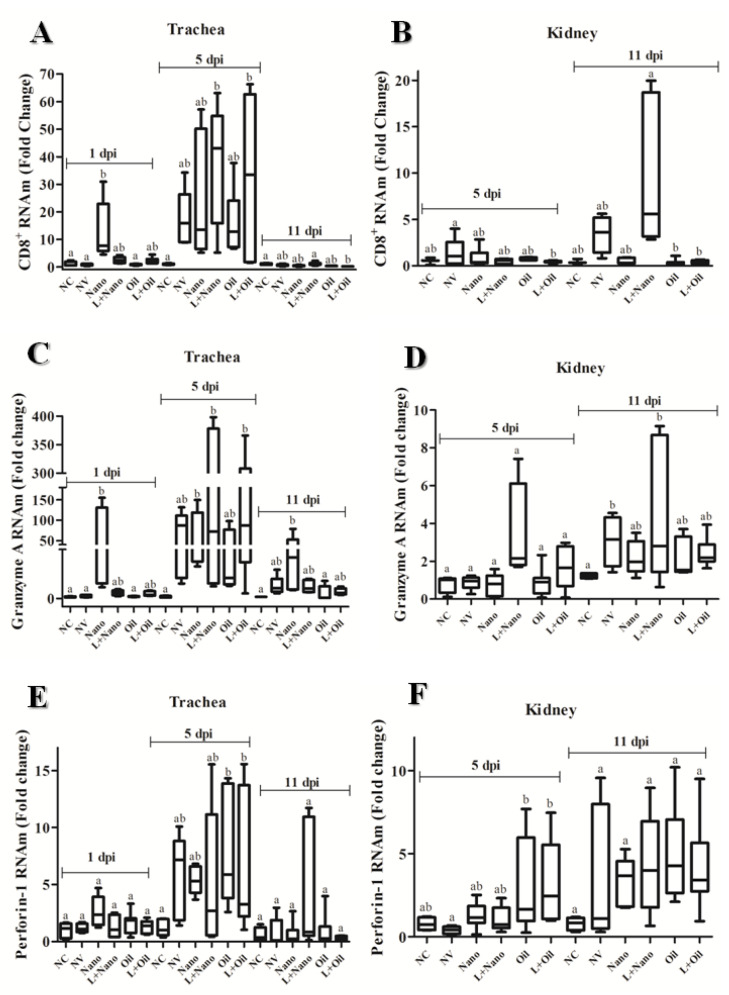
Means (± standard deviation) of relative expression of cell-mediated immune-response-related genes CD8+ mRNA expression (**A**,**B**); Granzyme A mRNA expression (**C**,**D**) and Perforin-1 mRNA expression (**E**,**F**) in trachea (**A**,**C**,**E**) and kidney (**B**,**D**,**F**) samples, observed at 1, 5, and 11 dpi of negative control (NC) group, nonvaccinated and challenged (NV) group, vaccinated with AvCoV-CS and challenged (Nano) group, vaccinated with H120 strain and AvCoV-CS and challenged (L+Nano) group, vaccinated with AvCoV-O and challenged (Oil) group, and vaccinated with H120 strain and AvCoV-O and challenged (L+Oil) group. Significantly (*p* ≤ 0.05) different values are shown as different letters.

The expression of genes related to activation of cytotoxic T lymphocytes (CD8β+, homologous Granzyme A, and Perforin-1) in tracheal and renal tissue samples from all the experimental groups is shown in Figure 2.

Increased expression of the CD8β gene was detected in the trachea from chickens of the Nano group at 1 dpi, which was significantly higher compared to the NC-, NV-, and Oil-vaccinated groups (*p* ≤ 0.05). At 5 dpi, the L+Nano- and L+Oil-vaccinated groups showed higher levels of CD8β transcripts compared to the NC group (*p* ≤ 0.05), while at 11 dpi, there was a decrease in the expression of this gene for these vaccinated groups. At this interval, significant differences were found between the NC and L+Nano groups compared to the L+Oil group (*p* ≤ 0.05). At 5 dpi, the expression of the CD8β gene in renal samples was higher for the NV group compared to the L+Oil group, but it was similar to those observed in other experimental groups, including the group of unvaccinated and unchallenged birds. At 11 dpi, there was an increase in the expression of the CD8β gene in the L+Nano group, which was higher than those found in the Oil- and L+Oil-vaccinated groups (*p* ≤ 0.05), while similar CD8β levels were observed in the NC, NV, and Nano groups (*p* ≥ 0.05).

Greater expression of the Granzyme A gene was observed in tracheal samples of the Nano group at 1 dpi compared to the NC, NV, and Oil groups (*p* ≤ 0.05). At 5 dpi, the Nano-, L+Nano-, and L+Oil-vaccinated groups showed significantly higher levels of expression of the Granzyme A gene compared to the NC group (*p* ≤ 0.05). At 11 dpi, decreased Granzyme A levels were found in the trachea of all challenged groups (vaccinated or not), but in the Nano group, higher levels were kept compared to the NC and Oil groups (*p* ≤ 0.05). The Granzyme A transcripts were markedly lower in the renal samples when compared to the trachea, and there was no significant difference at 5 dpi between experimental groups (*p* ≥ 0.05). At 11 dpi, chickens from the NV and L+Nano groups showed greater expression for this gene than the NC group (*p* ≤ 0.05).

There were no significant differences between groups in terms of the expression of the Perforin-1 gene in the trachea either at 1 or 11 dpi and in renal samples at 11 dpi (*p* ≥ 0.05). However, at 5 dpi, the Oil and L+Oil groups showed higher levels of Perforin-1 gene expression than the NC group (*p* ≤ 0.05). At 5 dpi, significantly higher expression of the Perforin-1 gene was also observed for the renal samples from the Oil and L+Oil groups than the NV group (*p* ≤ 0.05).

### 3.4. Evaluation of Mucosal and Systemic Anti-AvCoV Antibody Responses

**Figure 3:**
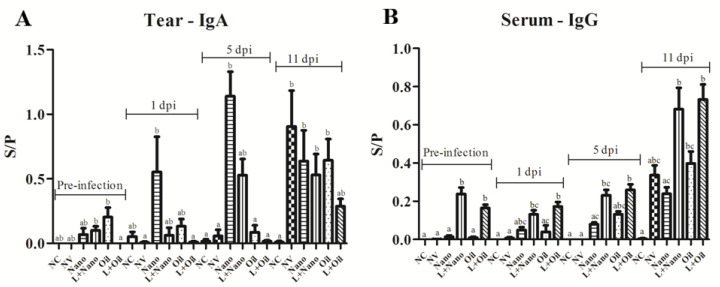
Means (± standard deviation) of mucosal IgA anti-AVCoV antibody levels (**A**) and systemic IgG anti-AVCoV antibody levels (**B**) anti-AvCoV titers for negative control (NC) group, nonvaccinated and challenged (NV), vaccinated with AvCoV-CS and challenged (Nano), vaccinated with H120 strain and AvCoV-CS and challenged (L+Nano), vaccinated with AvCoV-O and challenged (Oil), and vaccinated with H120 strain and AvCoV-O and challenged (L+Oil) groups. Significantly (*p* ≤ 0.05) different values are shown as different letters. S/P: sample-to-positive ratios.

The anti-AvCoV antibodies levels of the mucosal IgA isotype (lachrymal secretion) and systemic (blood serum) IgG during prechallenge and postchallenge intervals are shown in Figure 3. At the preinfection interval, there were no significant differences between vaccinated and challenged groups compared to the NV group. IgA levels increased at 1 dpi in chickens from the Nano group in relation to the NV- and L+Oil-vaccinated groups (*p* ≤ 0.05). In addition, IgA increased further in this group at 5 dpi compared to the NC, NV, Oil, and L+Oil groups. (*p* ≤ 0.05). At 11 dpi, levels of IgA antibodies remained high in the mucosa of chickens from the Nano-vaccinated group, and these antibodies also increased in the mucosa of the chickens from the NV-, L+Nano-, and Oil-vaccinated groups, showing significant differences in relation to the NC group (*p* ≤ 0.05).

Higher systemic IgG anti-AvCoV levels were found in the L+Nano and L+Oil groups compared to all other groups (*p* ≤ 0.05) at the preinfection interval. At 1 and 5 dpi, only the groups that were previously vaccinated with the attenuated AvCoV vaccine (L+Nano and L+Oil groups) presented significantly different levels compared to the NC and NV groups (*p* ≤ 0.05), while at 11 dpi, the Oil group exhibited higher IgG levels than the NC group.

### 3.5. Correlation between Parameters of the Immune Response against AvCoV Induced by Vaccines and the Pathological Changes Caused by This Virus

Correlations were determined and analyzed separately for groups that received the AvCoV vaccine encapsulated in chitosan nanoparticles (Nano and L+Nano groups) from those that received the oil adjuvanted vaccine (Oil and L+Oil groups). In general, the Nano- and L+Nano-vaccinated groups had a greater number of significant negative correlations between viral loads in tracheal and renal samples in terms of humoral and cellular immune responses (Table 3) than the other groups. Conversely, the chickens immunized with the oil adjuvant vaccine regime showed negative correlations, mainly with the levels of systemic anti-AvCoV IgG antibodies and the viral loads in tracheal and renal samples (Table 4).

## 4. Discussion

In the present study, chitosan nanoparticles used as carrier-adjuvant in an inactivated vaccine containing a BR-I strain of AvCoV were single administered or administered as a booster dose after the Mass attenuated vaccine and before challenge with the BR-I virulent strain. Both schemes of vaccination lead to enhanced immune responses and resulted in effective reduction of viral load and number of lesions in the trachea and kidney after challenge with BR-I AvCoV. The oily adjuvant in a similar formulation of this AvCoV strain inactivated vaccine administered via the intramuscular route afforded a similar protection status when preceded by a Mass attenuated vaccine, but the absence of complete protection was observed in the single Oil-vaccinated group.

Several factors can influence the type and intensity of the immune response induced by a viral vaccine, such as the antigen type (live attenuated or inactivated), the inclusion of carrier-adjuvants and their type, the vaccination strategy, the cells involved in antigen recognition, the age of the chicken and the route of administration [4,15,18,24,36].

Chickens single immunized with the chitosan nanoparticle vaccine containing an inactivated BR-I strain of AvCoV (Nano group) had earlier and higher levels of local IgA anti-AvCoV antibodies after challenge and higher expression levels of memory compared to CMI-related genes, such as the CD8β and Granzyme A genes in the trachea at 1 dpi. Chickens primed with the attenuated Mass vaccine followed by the AvCoV-CS vaccine (L+Nano) presented a stronger response of systemic IgG anti-AvCoV antibodies and higher levels of expression of CMI-related genes during the post-challenge period, characterized by the increased expression of the CD8β and Granzyme A genes in tracheal and renal samples at 5 and 11 dpi, respectively.

The results regarding the antibody and cellular immune responses corroborate with the findings of Lopes et al. [20], who described similar profiles of immune responses involving lachrymal IgA and IgG anti-AvCoV antibodies and IFN-γ gene expression in two groups of chickens immunized with the same inactivated vaccines and vaccination schedule adopted in this study. However, there is a paradoxical finding of the early and increased expression, at 1 dpi, of the CMI genes (CD8β and Granzyme A or IFN-γ genes) only in the group of chickens vaccinated with a single dose of inactivated chitosan nanoparticles vaccine (Nano group) compared to the group that was immunized with this vaccine preceded by the live attenuated Mass vaccine (L+Nano group). This may be attributed to the immune responses and the immune-mediators induced by primary vaccination of 1-day-old birds with the live attenuated vaccine. Thus, our hypothesis is that these immune responses may act through possible mechanisms of immunomodulation and regulation generated after booster vaccine dose in birds primarily immunized with the live attenuated vaccine. These mechanisms should be mediated mainly by antiviral IgG antibodies present in the mucosal and systemic compartments, as detected in the current study, whereas there are, at this time point, low or no IgG antibody levels in chickens immunized once with the chitosan nanoparticle inactivated vaccine. In fact, a similar event of negative immune modulation of CMI gene expression in the respiratory tract was reported by Guo et al. [37], after secondary immunization of chickens with the live attenuated AvCoV vaccine, leading the authors to suspect that there are high levels of IgG antiviral antibodies inhibiting and delaying the cellular immune responses in these secondarily immunized birds.

On the other hand, vaccination with a first dose of the attenuated Mass vaccine followed by the oil adjuvant BR-I AvCoV inactivated vaccine (L+Oil group) predominantly induced immune responses mediated by antibodies of the IgG isotype in the systemic compartment, as well as led to greater expression of Granzyme A, CD8β, and Perforin-1 genes in the trachea and the Perforin-1 gene in the kidney at 5 dpi. Interestingly, a previous study that adopted a similar vaccination schedule, reported significant anti-AvCoV IgG increases in both serum and lachrymal samples, as well as high expression of the CD8β chain and Granzyme homolog A in tracheal and renal samples at 3, 7, and 11 dpi of vaccinated chickens [24]. In addition, in this study, vaccinated chickens also presented a reduction in viral loads and histological lesions in both tracheal and renal samples after being challenged with a virulent strain. However, in our study, the group single immunized with the oil adjuvant AvCoV inactivated vaccine exhibited increased expression of the Perforin-1 gene in the trachea and kidney at 5 dpi, as well as slight changes in the anti-AvCoV antibody levels.

The immune-stimulating properties of chitosan nanoparticles as carrier-adjuvant in vaccines, including or no, other compounds such as saponin and mannose, and either for conventional viral antigen preparations or recombinant viral protein and nucleic acid formulations, have been demonstrated in several studies, especially with regard to their ability to induce local and systemic antibodies and T-cell immune responses against viral pathogens. This includes the induction of TH1/TH2 responses, especially when the vaccine is administered via the mucosa [12,13,15,16,19,26,38,39]. The main advantage of our developed AvCoV-CS vaccine is its capacity to safely activate immune responses mediated by antibody and T cells in the mucosal and systemic compartments, since the used antigen is an inactivated virus. Furthermore, our proposed vaccine may serve as a model for the formulation of other inactivated virus vaccines for poultry health for killed bacteria vaccines (bacterins) or for vaccines made of recombinant proteins of these pathogens, which can include some relevant advantages such as low cost and immune-potentiation for the vaccines containing this carrier-adjuvant [12,20,38].

Regarding the association of adjuvant Montanide ISA 71 and the inactivated AvCoV virus (AvCoV-O), the obtained results have not reached the same performance as that observed for the AvCoV-CS vaccine, since it was unable to induce strong humoral and cell-mediated immune responses and consequently led to incomplete protection after being challenged with single AvCoV-O administration. Nevertheless, studies using only this adjuvant for the avian influenza virus inactivated vaccine or its association with H6 virus-like particles have reported the induction of effective protection after challenge with this virulent virus [23,40]. Furthermore, Montanide ISA 71 has also been successfully used for vaccines consisting of Eimeria recombinant proteins and has induced efficient protection to infection with these pathogens [22,25]. In addition, the main change in CMI responses induced after immunization with a single dose of inactivated oil adjuvant vaccine was an upregulated expression of the Perforin-1 gene. This gene codes for a protein with enzymatic activity secreted by cytotoxic cells such as CD8 + T cells, T γδ cells, and natural killer cells (NK). The main role of this enzyme is to induce membrane pores in virus-infected cells for entry of Granzymes and other apoptotic-inducing factors into virus-infected target cells [41]. However, it should be considered that the putative immune-protective activity of Perforin-1 needs to be complemented by other mediators of the cytotoxic immune response, mainly the homologous Granzyme A and IFNγ, which if acting in concert, can control AvCoV infection more effectively [3,42].

The current study also demonstrated that the chickens that received the AvCoV-CS vaccine exhibited a significant negative correlation between the viral loads detected in the trachea and kidney and the humoral and cell-mediated immune responses, while a reduced degree of negative correlations was obtained in chickens vaccinated with oil the adjuvant inactivated AvCoV vaccine. Overall, these findings corroborated with those of previous studies, which found negative correlations between pathological changes caused by AvCoV and immune responses induced by attenuated or inactivated vaccines in chickens after infection with different virulent strains of AvCoV [3,20,24]. These findings highlight the fact that both humoral and cell-mediated immune memory responses are required for the reduction of virus load and pathological alterations after infection with virulent AvCoV [2,3].

## 5. Conclusions

The AvCoV-CS vaccine containing chitosan as a carrier-adjuvant administered via the mucosa (oculonasal route) induced early and high cellular and humoral immune responses to the BR-I AvCoV strain, especially at the mucosa of the respiratory tract when single administered or combined with the attenuated Mass heterologous vaccine, providing effective protection to the tracheal and renal tissues against a BR-I virulent strain. In contrast, the AvCoV inactivated vaccine associated with oil adjuvant Montanide ISA 71 (oil vaccine), administered as a single dose by the intramuscular route, did not induce protection against challenge with the virulent AvCoV strain, although the association of the oil adjuvant vaccine and the attenuated Mass vaccine induced similar protection compared to the groups that received the AvCoV-CS vaccine containing chitosan nanoparticles.

## Figures and Tables

**Table 1:** Experiment design.

Group	1st Day of Age	14th Day of Age	31st Day Of Age
NC	-	Nanoparticles with no virus	DMEM culture medium
NV	-	-	Challenged with AvCoV/Brazil/PR05 strain
Nano	-	Inactivated AvCoV-CS vaccine	Challenged with AvCoV/Brazil/PR05 strain
L+Nano	Attenuated H120 strain vaccine	Inactivated AvCoV-CS vaccine	Challenged with AvCoV/Brazil/PR05 strain
Oil	-	Inactivated AvCoV-O vaccine	Challenged with AvCoV/Brazil/PR05 strain
L+Oil	Attenuated H120 strain vaccine	Inactivated AvCoV-O vaccine	Challenged with AvCoV/Brazil/PR05 strain

Groups (NC: chickens nonvaccinated and nonchallenged; NV: chickens nonvaccinated and challenged; Nano: chickens vaccinated with AvCoV-CS via oculonasal route; L+Nano: chickens vaccinated with H120 attenuated vaccine and AvCoV-CS, both via oculonasal route; Oil: chickens vaccinated with AvCoV-O by intramuscular route; L+Oil: chickens vaccinated with H120 attenuated vaccine via oculonasal route and AvCoV-O by intramuscular route). Vaccines (AvCoV-CS: inactivated AvCoV vaccine encapsulated in chitosan nanoparticles; AvCoV-O: incorporated into the adjuvant Montanide ISA 71; L: H120 attenuated vaccine).

**Table 2:** Means (± standard deviation) of microscopic lesion scores in tracheal and renal samples observed in NC, NV, Nano, L+Nano, Oil, and L+Oil groups at 5 and 11 dpi.

Group	Trachea	Kidney
NC	0.0 ± 0.0 ^a^	0.0 ± 0.0 ^a^
NV	1.241 ± 1.331 ^bd^	0.9778 ± 0.9412 ^b^
Nano	0.8000 ± 0.9467 ^bc^	0.2222 ± 0.5520 ^ac^
L+Nano	0.5526 ± 0.8390 ^c^	0.5167 ± 0.7700 ^c^
Oil	1.575 ± 1.156 ^d^	0.8889 ± 0.9000 ^b^
L+Oil	0.9375 ± 1.194 ^bc^	0.5556 ± 0.9801 ^bc^

Groups (NC: chickens nonvaccinated and nonchallenged; NV: chickens nonvaccinated and challenged; Nano: chickens vaccinated with AvCoV-CS via oculonasal route; L+Nano: chickens vaccinated with H120 attenuated vaccine and AvCoV-CS, both via oculonasal route; Oil: chickens vaccinated with AvCoV-O by intramuscular route; L+Oil: chickens vaccinated with H120 attenuated vaccine via oculonasal route and AvCoV-O by intramuscular route). Vaccines (AvCoV-CS: inactivated AvCoV vaccine encapsulated in chitosan nanoparticles; AvCoV-O: incorporated into the adjuvant Montanide ISA 71; L: H120 attenuated vaccine). Significantly (*p* ≤ 0.05) different values are shown with different letters in each column.

**Table 3:** Correlation coefficients between results related to immune response at 5 dpi and pathological changes in chickens vaccinated with AvCoV-CS and the challenged group.

	IgA	IgG	CD8β	Granzyme	Perforin
Viral load 5 dpi (T)	−0.499 *	−0.366	−0.323	−0.553 *	−0.147
Viral load 11 dpi (T)	−0.413 *	−0.487 *	−0.245	−0.551 *	-
Viral load 5 dpi (K)	-	−0.533 *	-	-	−0.508
Viral load 11 dpi (K)	-	−0.181	-	−0.517 *	-

dpi: days post infection; T: tracheal; K: kidney; * (asterisk) represents a significant correlation by Spearman’s correlation test with a 95% confidence interval.

**Table 4:** Correlation coefficients between results related to immune response at 5 dpi and pathological changes in chickens vaccinated with AvCoV-O and the challenged group.

	IgA	IgG	CD8β	Granzyme	Perforin
Viral load 5 dpi (T)	-	−0.148	−0.058	-	-
Viral load 11 dpi (T)	-	−0.419 *	-	-	-
Viral load 5 dpi (K)	-	−0.438 *	-	−0.122	−0.339
Viral load 11 dpi (K)	-	−0.018		−0.310	−0.024

dpi: days post infection; T: tracheal; K: kidney; * (asterisk) represents a significant correlation by Spearman’s correlation test with a 95% confidence interval.

## Data Availability

Data are not publicly available due to privacy, but can be obtained upon justified request to the corresponding author.

## Supplementary Materials

The following are available online at https://www.mdpi.com/article/10.3390/vaccines9121457/s1. Figure S1. (**A**) Microscopic changes observed in tracheal samples: 1—loss of cilia (deciliation) and loss of epithelial cells, 2—presence of lymphocytic cells infiltrates, 3—congestion. (**B**) Mononuclear inflammation was observed in renal tissues (black arrow).
