# Supplementary figures and images for: Comparative Evaluation of Immune Responses and Protection of Chitosan Nanoparticles and Oil-Emulsion Adjuvants in Avian Coronavirus Inactivated Vaccines in Chickens
Source: Vaccines (Basel). 2021 Dec 9;9(12):1457. doi: 10.3390/vaccines9121457 (PMC8705532; doi:10.3390/vaccines9121457)

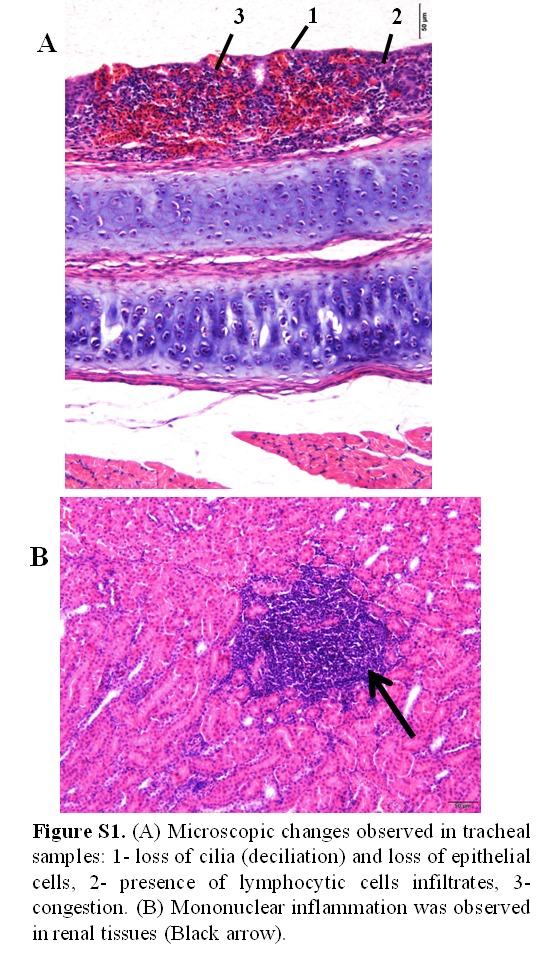

Supplement: Supplementary file 1 [file vaccines-09-01457-s001.zip › vaccines-1460206-supplementary.tif]
